# Safe Performance of Track Dilation and Bile Aspiration with ERCP Catheter in EUS-Guided Hepaticogastrostomy with Plastic Stents: A Retrospective Multicenter Study

**DOI:** 10.3390/jcm11174986

**Published:** 2022-08-25

**Authors:** Ikuhiro Kobori, Yusuke Hashimoto, Taro Shibuki, Kei Okumura, Masanari Sekine, Aki Miyagaki, Yoshihiro Sasaki, Yuichi Takano, Yasumi Katayama, Masaru Kuwada, Yoshinori Gyotoku, Yumi Kusano, Masaya Tamano

**Affiliations:** 1 Department of Gastroenterology, Dokkyo Medical University Saitama Medical Center, Koshigaya 343-8555, Japan; 2 Department of Hepatobiliary and Pancreatic Oncology, National Cancer Center Hospital East, Kashiwa 277-8577, Japan; 3 Department of Gastroenterology, Jichi Medical University Saitama Medical Center, Saitama 330-0834, Japan; 4 Department of Gastroenterology, Toyooka Hospital, Toyooka 668-8501, Japan; 5 Department of Gastroenterology, National Organization Disaster Medical Center, Tokyo 190-0014, Japan; 6 Department of Gastroenterology, Fujigaoka Hospital, Showa University, Yokohama 227-8501, Japan; 7 Endoscopy Center, Dokkyo Medical University Saitama Medical Center, Koshigaya 343-8555, Japan

**Keywords:** endoscopic ultrasonography, endoscopic-ultrasound-guided biliary drainage, endoscopic-ultrasound-guided hepaticogastrostomy, bile aspiration

## Abstract

Objectives: Endoscopic-ultrasound-guided hepaticogastrostomy (EUS-HGS) with plastic stent placement is associated with a high incidence of adverse events that may be reduced using an endoscopic retrograde cholangiopancreatography (ERCP) contrast catheter in the track dilation step. In this study, we evaluated the usefulness of track dilation and bile aspiration performed with an ERCP contrast catheter in EUS-HGS with plastic stent placement. Methods: In a multicenter setting, 22 EUS-HGS cases dilated with an ERCP contrast catheter were analyzed retrospectively and compared between a bile aspiration group and no bile aspiration group. Results: Overall, adverse events occurred in three (13.6%) cases of bile leakage, three (13.6%) cases of peritonitis, and one (4.5%) case of bleeding. Comparing patients with and without bile aspiration, 6 of the 11 patients (54.5%) with no bile aspiration had adverse events, whereas only 1 of the 11 patients (9.1%) who had bile aspiration, as much bile as possible, had an adverse event (bleeding). In univariate analysis, the only factor affecting the occurrence of adverse events was bile aspiration whenever possible (odds ratio, 12.0; 95%CI 1.12–128.84). Conclusions: In EUS-HGS with plastic stent placement, track dilation and bile aspiration with an ERCP contrast catheter may be useful in reducing adverse events.

## 1. Introduction

The endoscopic-ultrasound-guided biliary drainage (EUS-BD) procedure has become widely used in recent years [1,2,3,4,5] but no consensus has yet been established regarding aspects such as puncture site, type of dilator, and type of stent [6,7,8]. Endoscopic-ultrasound-guided hepaticogastrostomy (EUS-HGS) is an EUS-BD procedure that comprises four steps: bile duct puncture, guidewire passage, track dilation, and stent placement. The occurrence of postoperative adverse events such as bile leakage following EUS-HGS is more frequent in cases with the placement of a plastic stent than in cases where a self-expandable metallic metal stent (SEMS) is used [9]. In the case of an SEMS, even if the track dilation is greater than the outer diameter of the sheath before stent deployment, the stent will eventually expand beyond the dilation diameter and thus prevent bile leakage, whereas a plastic stent is more likely to leak bile when the track is dilated beyond the stent diameter. Therefore, when using plastic stents in EUS-HGS, it is preferable to use a bougie dilator, which dilates equivalently to the stent diameter, rather than a balloon catheter, which dilates beyond the stent diameter. The usefulness of bile aspiration prior to stent placement during EUS-HGS, especially bile aspiration of 10 mL or more, has recently been reported [10]. Although most of the stents in that report were SEMSs, bile aspiration may also be desirable in EUS-HGS with plastic stent placement. To aspirate a large amount of bile, it is necessary to dilate the bile duct with a bougie dilator and then place a regular endoscopic retrograde cholangiopancreatography (ERCP) contrast catheter into the bile duct for aspiration. However, if the pressure in the bile duct remains high, leakage of bile can be expected between dilation and placement of the regular ERCP contrast catheter. Ideally, bile would be aspirated directly using the bougie dilator, but its very thin tip, designed to facilitate tracking, [11] makes it unsuitable for aspiration. One solution to this problem is to use an ERCP contrast catheter. The use of a regular ERCP contrast catheter as the dilation device may reduce procedure time and cost as well as further reduce the risk of biliary leakage, as it eliminates the need to insert a bougie dilator or other dilation device. In this study, we investigated the usefulness of using an ERCP contrast catheter for dilatation and bile aspiration in EUS-HGS with plastic stent placement.

## 2. Materials and Methods

### 2.1. Study Design

**Figure 1:**
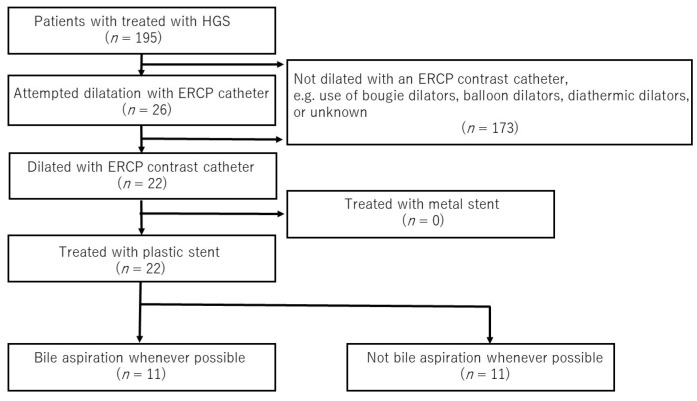
Flowchart of the study design. Abbreviations: ERCP, endoscopic retrograde cholangiopancreatography; HGS, hepaticogastrostomy.

**Figure 2:**
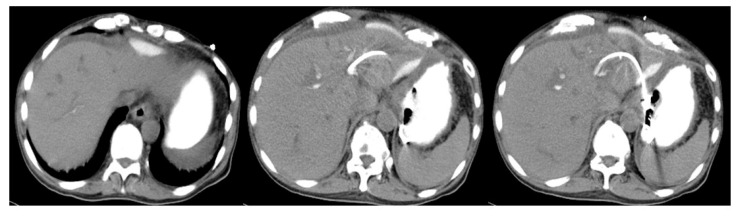
CT scan image showing bile leak. Contrast medium is filling the stomach. Leakage of contrast medium can be seen near the stenting site.

This was a retrospective multicenter study of patients with biliary obstruction who had undergone EUS-HGS between April 2015 and December 2021 at any of six participating facilities in Japan. The study was approved by the review board at each respective institution and was conducted in accordance with the tenets of the Helsinki Declaration. EUS-HGS was performed according to the indications at each institution, and we retrospectively reviewed patients who underwent track dilation with an ERCP contrast catheter and plastic stent placement (Figure 1). The primary outcome was the rate of adverse events after plastic stent placement. The secondary outcomes were the cause of recurrent biliary obstruction (RBO), time to recurrent biliary obstruction (TRBO), and functional success. RBO was defined as the recurrence of obstructive jaundice and/or cholangitis due to stent occlusion or migration. TRBO was defined as the length of time between stent placement and the occurrence of RBO. Functional success was defined as: (1) a 50% decrease in or normalization of the serum total bilirubin level within 14 days of stent placement; (2) in the case of cholangitis without elevation of the serum total bilirubin level, an improvement of cholangitis. Adverse events other than RBO were categorized as post-procedure complications, which included bile leakage, peritonitis, bleeding, perforation, pancreatitis, cholecystitis, aspiration pneumonia, liver abscess, mediastinal emphysema, and pneumoperitoneum. These complications were categorized as early (within 30 days) or late (at 31 days or later). The time point of adverse events was defined as the point when symptoms associated with these conditions were observed. The adverse events were classified and graded according to the American Society for Gastrointestinal Endoscopy Workshop reports [12]. Peritonitis was diagnosed on the basis of clinical peritoneal inflammation. Bile leakage was defined as the patient presenting with new fluid collection around the EUS-HGS stent outside the stomach and the liver, confirmed by CT (Figure 2).

The study also examined adverse events categorized into two groups: with bile aspiration (as much bile as possible was aspirated) and without bile aspiration (bile was not aspirated prior to plastic stenting). Bile aspiration was performed only at specific study centers. Regardless of the degree of bile duct dilatation (total bilirubin level), bile aspiration was performed starting at a certain date with the expectation of reducing complications.

### 2.2. Patients

Of all patients with biliary obstruction who had undergone EUS-HGS between April 2015 and December 2021, 22 were treated with track dilation by an ERCP contrast catheter and placement of a plastic stent. Biliary obstruction was diagnosed based on the clinical, laboratory, radiographic, and pathological findings. Distal biliary obstruction was defined as a site of stenosis at least 2 cm away from the liver hilum. EUS-HGS was indicated for patients in whom it was difficult to reach the papilla due to gastrointestinal obstruction or surgically altered anatomy, and those in whom it was difficult to cannulate the bile duct even if the papilla was reached. Cases of benign as well as malignant disease were included. Patients who underwent biliary drainage as well as initial drainage were included. Performance status refers to the Eastern Cooperative Oncology Group performance status [13].

### 2.3. Procedures

**Figure 3:**
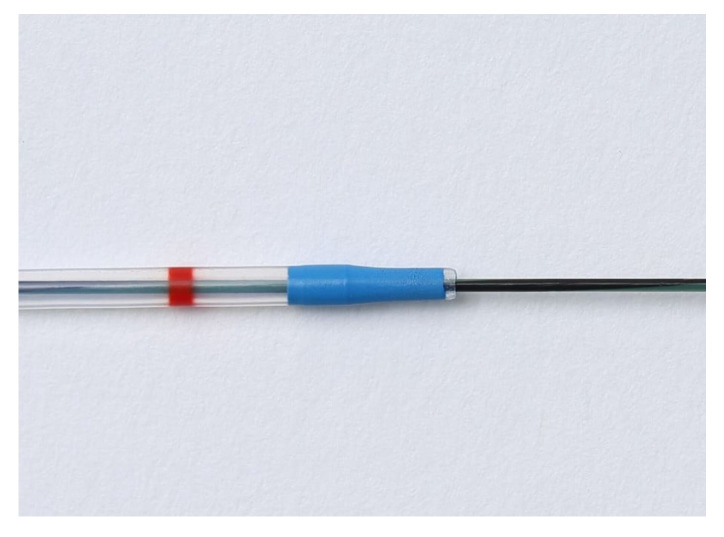
Tapered-tip single-lumen ERCP contrast catheter.

**Figure 4:**
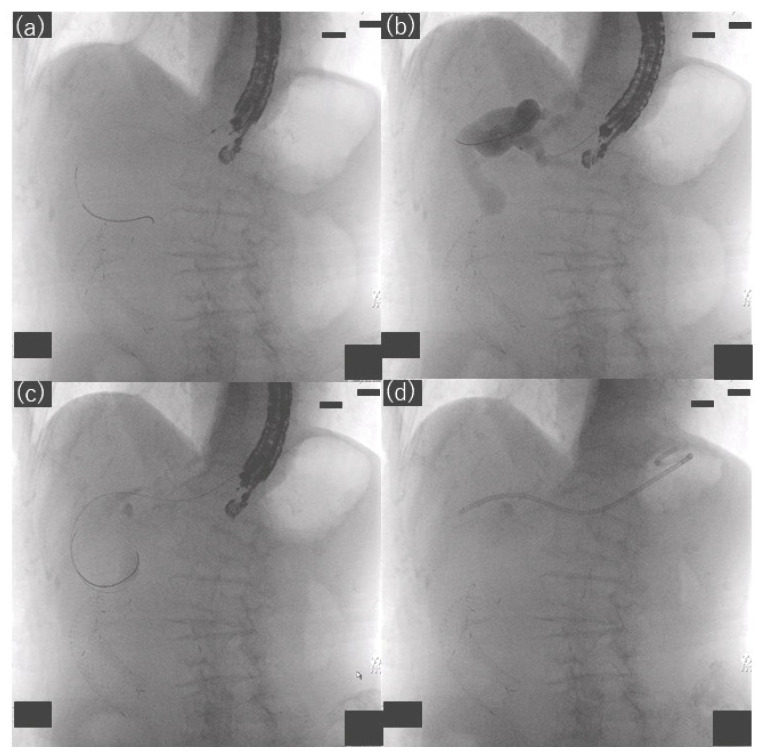
Fluoroscopic images showing hepaticogastrostomy. (**a**) Bile duct puncture and guidewire passage, (**b**) track dilation and cholangiography with an ERCP contrast catheter, (**c**) bile aspiration with an ERCP contrast catheter, (**d**) stent placement.

**Figure 5:**
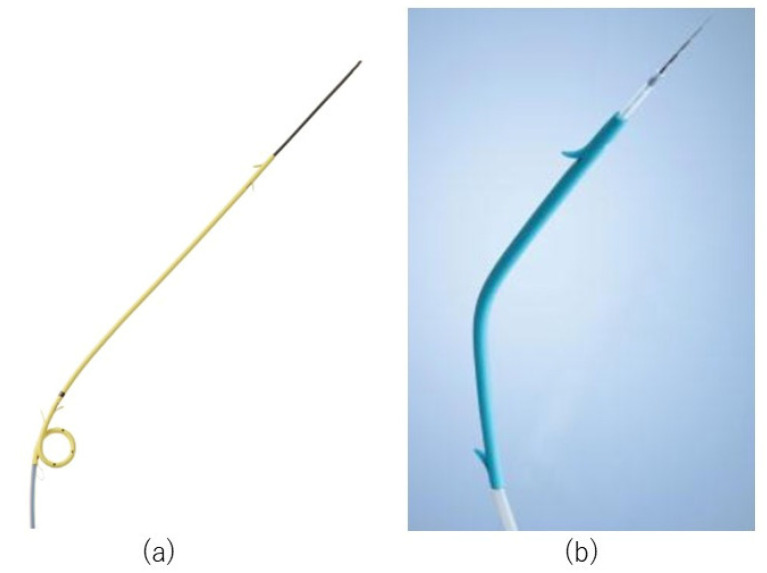
Plastic stent. (**a**) Through & Pass Type-IT (7-Fr, 14 cm, Gadelius Medical, Tokyo, Japan). (**b**) Quick Place V (7-Fr, Olympus Medical Systems, Tokyo, Japan).

We used a linear echoendoscope for EUS-HGS (GF-UCT 240 or GF-UCT 260, Olympus Medical Systems, Tokyo, Japan; EG 580 UT, Fujifilm Corp., Tokyo, Japan). The left lobe of the liver was observed by echoendoscope, and the intrahepatic bile duct was punctured and contrast-enhanced using a 19- or 22-gauge needle. After placement of a guidewire in the bile duct, a tapered-tip single-lumen ERCP contrast catheter (ERCP Catheter, 1.6~2.3 mm, MTW Endoskopie, Wesel, Germany) was introduced to dilate the track, and finally a plastic stent was placed (Figure 3 and Figure 4). In cases where bile was aspirated, the bile was aspirated as much as possible after track dilation with an ERCP catheter, and often more than 20 mL was aspirated. We performed cholangiography after aspirating the bile as much as possible. Then, except for hilar stenosis, after cholangiography was performed, aspiration was performed again until there was no more contrast media, and then a stent was placed. In a case of hilar stenosis, cholangiography was performed, followed by another small volume aspiration, and a stent was placed with contrast remaining. Two types of plastic stents were used: Through & Pass Type-IT (7-Fr, 14 cm, Gadelius Medical, Tokyo, Japan; Figure 5a) and Quick Place V (7-Fr, 11 cm, 15 cm, Olympus Medical Systems, Tokyo, Japan; Figure 5b). The stent length was determined by the cholangiographic findings. Procedure time was calculated from insertion of the echoendoscope into the mouth to its removal. If the track was difficult to dilate with an ERCP contrast catheter, a bougie dilator (ES Dilator, Zeon Medical Co., Tokyo, Japan) was used [11], and the rate of bougie dilator use was additionally studied. Antegrade stent placement was performed using an uncovered SEMS or a plastic stent prior to EUS-HGS stent placement. The decision regarding antegrade stent placement was at the discretion of the endoscopist.

### 2.4. Follow-Up

Patients were followed up at each facility until death or until the date of last known survival; or in the case of benign disease, until the cause of the benign disease was corrected and the EUS-HGS route stent was no longer needed and removed. Computed tomography was performed when adverse events were suspected based on the clinical symptoms or blood tests.

### 2.5. Statistical Analysis

All statistical analyses were performed using StatFlex Ver. 6 (Artec Inc., Osaka, Japan). TRBO was estimated using the Kaplan–Meier method. Patients who had stent removal or died without RBO were treated as censored cases. Factors affecting adverse events were assessed using univariate logistic regression analysis. Candidate factors affecting adverse events included age, performance status, puncture site, preoperative cholangitis, bile aspiration, antegrade stent placement, and procedure time. *p* < 0.05 was considered to indicate statistical significance.

## 3. Results

### 3.1. Patient Characteristics

The patient characteristics are summarized in Table 1. The site of biliary obstruction was distal in 14 patients (63.6%), hilar in 5 patients (22.7%), and anastomotic in 3 patients (13.6%). Four patients (18.2%) had cholangitis prior to the EUS-HGS procedure.

### 3.2. Outcomes

Table 2 summarizes the procedure outcomes, and Table 3 summarizes the clinical outcomes. Regarding aspiration of bile prior to plastic stent placement, bile was not aspirated in 11 patients (50.0%), and as much bile as possible was aspirated in 11 patients (50.0%). A conventional dilatation device was used in four patients in whom track dilation was difficult using an ERCP contrast catheter. Track dilation was achieved using an ERCP contrast catheter in 22 of the 26 patients (84.6%).

Functional success was achieved in 20 patients (90.9%). The median duration of observation was 68.5 (range, 12–610) days, and 18 patients (81.8%) died during the observation period. The cause of death was progression of the primary disease in 17 patients and a cause other than the primary disease in 1 patient. No death was attributed to EUS-HGS.

**Figure 6:**
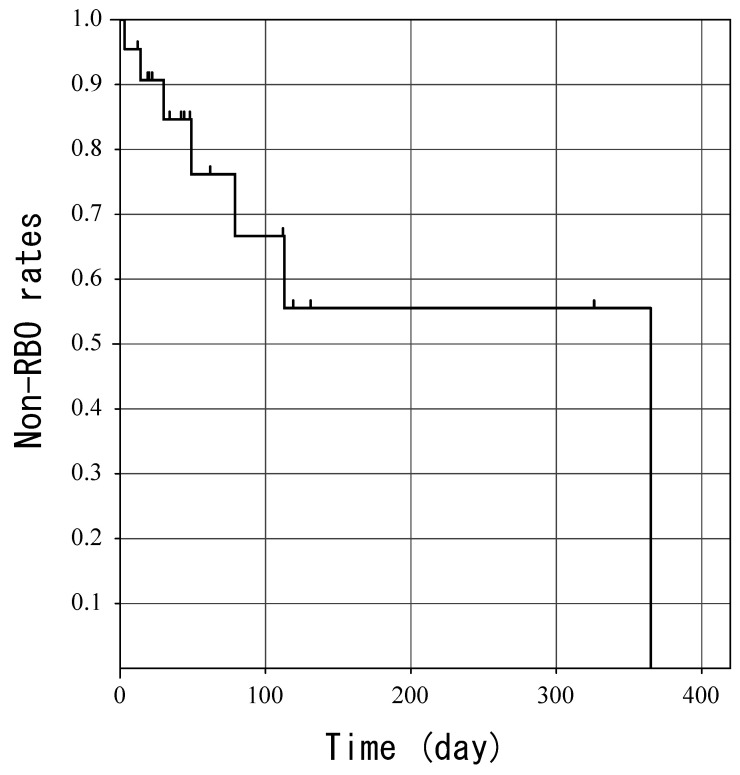
Kaplan–Meier curve of time to recurrent biliary obstruction. The small vertical bars indicate censored patients.

RBO was observed in seven patients (35.0%). The cause of RBO was sludge formation in four patients, migration (toward the gastric side) in one patient, and another cause in two patients. Two patients underwent stent exchange on days 112 and 119, prior to the onset of RBO, and were treated as censored for statistical purposes. Median TRBO was 365 (range, 3–382) days (Figure 6).

Adverse events other than RBO included bile leakage in three patients (13.6%), peritonitis in three patients (13.6%), and bleeding in one patient (4.5%), all of which were mild, occurred early, and were relieved by conservative treatment. In a comparison of patients with and without bile aspiration prior to plastic stenting, 6 of the 11 patients (54.5%) who did not have bile aspiration had adverse events, whereas only 1 of the 11 patients (9.1%) who had bile aspiration, as much bile as possible, had an adverse event (bleeding). Table 4 summarizes the patient characteristics and outcomes with and without bile aspiration whenever possible. Univariate analyses were performed for factors affecting adverse events (Table 5). Bile aspiration, as much bile as possible, was associated with the occurrence of AEs in the univariate analysis.

## 4. Discussion

Although the use of plastic stents in EUS-HGS has been reported to carry risk for bile leakage [9], the results of our study suggest that biliary aspiration prior to plastic stent placement may considerably reduce the risk of bile leakage and other adverse events. The usefulness of biliary aspiration in EUS-HGS has been reported [10]. The paper reported that biliary aspiration of 10 mL or more during HGS reduces adverse events, and the stents used were metallic stents in 70% and plastic stents in 30% of cases. We believe we have demonstrated that similar results can be obtained even when limited to EUS-HGS with plastic stents. If bile aspiration is performed with an ERCP contrast catheter after track dilation when intraductal biliary pressure is high, bile may leak out during device exchange, and, therefore, the timing of bile aspiration should be simultaneous with track dilation. However, as bile aspiration is difficult to achieve with specialized dilatation devices or balloon catheters with tapered tips, it makes the most sense to use an ERCP contrast catheter that can dilate the track and aspirate bile at the same time. Although it is possible to aspirate bile with a puncture needle prior to track dilation, it is anticipated that a large amount of bile aspiration will cause the bile duct to narrow, which may cause the puncture needle to be pulled out of the bile duct and subsequent guidewire insertion to be difficult. In the present study, we were able to dilate the track and aspirate bile with the ERCP contrast catheter in 84.6% of patients, which is considered a good result. It is worth attempting this technique before using a dedicated fistula-dilating device. We are working to improve tracking of the ERCP contrast catheter using new catheters with a firm, straight tip and by the operator applying slight tension to the guidewire when tracking with the ERCP contrast catheter. In addition, the bile duct should be punctured from B2 whenever possible, as this allows the guidewire and instruments to be inserted in a straight line and improves tracking performance. In the present study, puncture was from B2 in 15 patients (68.2%), which is a large number.

Unlike metal stents, plastic stents do not shorten. There are also plastic stents available for EUS-HGS with pigtail-type stent ends on the gastric side [14], which minimizes the possibility of serious adverse events such as gastric stent end migration into the abdominal cavity [15]. In the present study, there was no instance of gastric stent end migration into the abdominal cavity. Shorter stent patency is a concern in EUS-HGS with plastic stents compared to metal stents [1,16]. In the majority of cases, however, the stent occlusion occurs when the fistula has already formed, and unlike metal stents with uncovered stent ends on the liver side [17], the stent can be removed and replaced with a new stent in the event of stent occlusion. Even in the event of stent occlusion, it is often not a clinical or technical problem, as the stent can be safely replaced. In addition, as bile aspiration during EUS-HGS may significantly control adverse events, it appears that EUS-HGS with a plastic stent is suitable for the purpose of ensuring safe fistula formation. In patients who undergo transpapillary bile duct stenting using the usual ERCP technique, periodic stent replacement before RBO is often performed and is considered useful in actual clinical practice, especially in cases of hilar stenosis. In the EUS-HGS route, periodic replacement of the plastic stent before RBO may be also an option.

EUS-HGS using metal stents with a thin delivery system that eliminates the track dilation procedure has recently been reported in many cases [18] and is expected to reduce the adverse events associated with shorter examination time. However, it is difficult to perform bile aspiration before stenting with this technique and caution is required, especially in cases of cholangitis, because undrained infected bile may remain and cause prolonged cholangitis if the metal stenting results in obstruction of the bile duct regional branch [19]. Based on the present results, we believe that stent placement after bile aspiration is desirable. However, track dilation with an ERCP contrast catheter was difficult in a small number of our cases, and we hope that a dedicated EUS-HGS device that facilitates track dilation and allows bile aspiration will be introduced in the future.

There are several limitations to this study, including its small sample size and retrospective study design. Therefore, selection bias may not be excluded. Some adverse events may have been missed because CT was not performed in all patients on the day after EUS-HGS. The exact amount of bile aspirated is unknown because there is no accurate description of the amount of bile aspirated. It is impossible to give a clear figure for how much should be aspirated, which may reduce reproducibility. In addition, the success rate of track dilation by ERCP contrast catheter may be lower than the present results because a dedicated fistula dilation device might have been used from the beginning if we had expected that track dilation by ERCP contrast catheter would be difficult. Further verification in randomized controlled trials is required in the future.

## 5. Conclusions

In EUS-HGS with plastic stent placement, track dilation and bile aspiration with an ERCP contrast catheter may be useful in reducing adverse events. Although it cannot be applied to all cases, it can be applied to quite a large number of cases, and we believe it is worth a try.

## Figures and Tables

**Table 1:** Patient characteristics.

Characteristic	Value
Age, y	72 (47–90)
Sex, male/female	12/10
Cause of biliary obstruction	9 (40.9)
Gastric cancer	
Pancreatic cancer	6 (27.3)
Bile duct cancer	3 (13.6)
Duodenal cancer	2 (9.1)
Intrahepatic gallstone	1 (4.5)
Stenosis of choledochojejunostomy	1 (4.5)
Performance Status	
0	6 (27.3)
1	9 (40.9)
2	2 (9.1)
3	5 (22.7)
Site of biliary obstruction	
Distal	14 (63.6)
Hilar	5 (22.7)
Anastomotic	3 (13.6)
Indication of EUS-HGS	
Difficulty reaching the papilla	12 (54.5)
Surgically altered anatomy	7 (31.8)
Difficulty cannulating the bile duct	3 (13.6)
Presence of cholangitis before EUS-HGS	4 (18.2)

*n* = 22. Data are expressed as the median (range) or number (%). Continuous variables are presented as the median (range), categorical variables as the absolute number (percentage). Abbreviation: EUS-HGS, endoscopic-ultrasound-guided hepaticogastrostomy.

**Table 2:** Procedure outcomes.

	Value
Procedure time (min)	45.5 (15–90)
Puncture needle used	
19-gauge	21 (95.5)
22-gauge	1 (4.5)
Puncture site	
B2	15 (68.2)
B3	7 (31.8)
Types of plastic stents	
Through & Pass Type-IT	19 (86.4)
Quick Place V	3 (13.6)
Antegrade stent placement	4 (18.2)
Bile aspiration whenever possible	
No	11 (50)
Yes	11 (50)

*n* = 22. Data are expressed as the median (range) or number (%). Continuous variables are presented as the median (range), categorical variables as the absolute number (percentage).

**Table 3:** Clinical outcomes.

	*n* (%)
Functional success	20 (90.9)
Recurrent biliary obstruction	7 (31.8)
Occlusion	6 (27.3)
Sludge formation	4 (18.2)
Other	2 (9.1)
Migration (toward the gastric side)	1 (4.5)
Adverse events other than recurrent biliary obstruction	
Early adverse events	7 (31.8)
Bile leakage	3 (13.6)
Peritonitis	3 (13.6)
Bleeding	1 (4.5)
Late adverse events	0 (0)

*n* = 22.

**Table 4:** Patient characteristics and outcomes with and without bile aspiration whenever possible.

Bile Aspiration Whenever Possible	Yes (*n* = 11)	No (*n* = 11)
Preprocedural cholangitis (*n*)	3	1
Procedure time (min)	47 (27–89)	42 (15–90)
Time that the bile aspiration takes (min)	13 (5–20)	
Antegrade stent placement (*n*)	2	2
Adverse events other than recurrent biliary obstruction (*n*)	1	6
Bile leakage (*n*)	0	3
Peritonitis (*n*)	0	3
Bleeding (*n*)	1	0

Continuous variables are presented as the median (range), categorical variables as the absolute number.

**Table 5:** Factors affecting adverse events.

Univariate Analysis OR (95%CI)		*p* Value
Age (years)		0.29
<75	1	
≥75	2.86 (0.42–19.65)	
Performance status		0.25
0–1	1	
2–4	0.46 (0.02–2.67)	
Puncture site		0.45
B2	1	
B3	0.48(0.07–3.19)	
Preprocedural cholangitis		0.75
Yes	1.50 (0.13–17.67)	
No	1	
Bile aspiration whenever possible		0.04
No	12.0 (1.12–128.84)	
Yes	1	
Antegrade stent placement		0.40
Yes	2.60 (0.28–23.81)	
No	1	
Procedure time (min)		0.65
<44.5	1	
≥44.5	1.52 (0.25–9.29)	

Abbreviations: OR, odds ratio; CI, confidence interval.

## Data Availability

Not applicable.
